# Fine‐scale spatial variation in eastern grey squirrel melanism across a complex urban landscape

**DOI:** 10.1111/1365-2656.70295

**Published:** 2026-07-22

**Authors:** Francis Quang‐Hai Dinh, Isabella C. Richmond, Andrew P. Hendry, Allison M. Roth

**Affiliations:** ^1^ Department of Biology McGill University Montreal Quebec Canada; ^2^ Department of Biology Concordia University Montreal Quebec Canada; ^3^ Division of Biological Sciences University of Missouri–Columbia Columbia Missouri USA; ^4^ Groupe de Recherche Interuniversitaire en Limnologie (GRIL) Université de Montréal Montreal Quebec Canada

**Keywords:** eastern grey squirrel, landscape ecology, melanism, phenotypic spatial variation, river barriers, urban adaptation, urban evolution, urban–rural clines

## Abstract

1. Urban environments impose novel selective pressures that can drive rapid phenotypic and genetic change in wild populations. While previous studies have focused on broad urban–rural contrasts or cross‐city comparisons, less is known about how phenotypic traits vary across fine spatial scales within cities, where environmental gradients and geographic features may create localized heterogeneity.
2. We studied spatial variation in coat colour morph of the eastern grey squirrel (*Sciurus carolinensis*) across the Montreal Metropolitan Area, focusing on grey and melanic morphs, the latter of which is caused by an incompletely dominant MC1R mutation. We conducted standardized scan sampling surveys across 197 parks and supplemented this data with citizen science observations from iNaturalist and SquirrelMapper.
3. As an archipelago segmented by major waterways, Montreal exhibits a spatial mosaic where phenotypic gradients are partitioned by physical barriers. We examined variation in melanism in relation to urbanization, local environmental conditions (e.g. temperature and canopy cover) and geographic structure.
4. Unlike patterns in many cities where melanism peaks in urban cores, melanism in Montreal was generally negatively correlated with urbanization, but the strength and direction of urban–rural clines varied sharply across the city's complex island landscape. Abrupt shifts in morph frequency corresponded with major waterways, including the Saint‐Lawrence River and its tributaries, suggesting that rivers might act as partial barriers to dispersal which partition the landscape into distinct phenotypic regions.
5. Consistent with the thermoregulatory advantages of melanism, melanic morph frequency increased most in areas with cooler winter temperatures and higher canopy cover. Structural equation modelling revealed that urbanization indirectly suppresses melanism by altering these two variables.
6. Our results suggest that phenotypic patterns within cities are not uniform but are instead shaped by fine‐scale environmental heterogeneity and geographic barriers, highlighting the need to move beyond simple urban–rural contrasts when predicting phenotypic variation in urban populations.

## Graphical abstract

By sampling 197 parks, the study demonstrates how natural geography creates divergence within cities. The findings reveal that the St. Lawrence River and its tributaries act as barriers, disrupting urban melanism clines in grey squirrels. Ultimately, the research indicates that natural features still dictate evolutionary rules in anthropogenic landscapes.

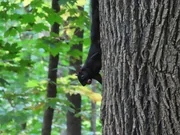

## Résumé

1. Les environnements urbains exercent des pressions de sélection inédites qui peuvent entraîner des changements phénotypiques et génétiques rapides au sein des populations sauvages. Bien que les études antérieures se soient concentrées sur des contrastes généraux entre milieux urbains et ruraux ou sur des comparaisons entre différentes villes, on sait encore peu de choses sur la manière dont les traits phénotypiques varient à de fines échelles spatiales au sein même des villes, où les gradients environnementaux et les caractéristiques géographiques peuvent générer une hétérogénéité localisée.
2. Nous avons étudié la variation spatiale de couleur du pelage de l'écureuil gris de l'Est (Sciurus carolinensis) à travers la région métropolitaine de Montréal, en nous concentrant sur les morphes gris et mélaniques, ce dernier étant causé par une mutation du gène MC1R à dominance incomplète. Nous avons mené des relevés standardisés par échantillonnage instantané dans 197 parcs et avons complété ces données par des observations issues de la science citoyenne, via les plateformes iNaturalist et SquirrelMapper.
3. En tant qu'archipel délimité par d'importants cours d'eau, Montréal présente une mosaïque spatiale dans laquelle les gradients phénotypiques sont compartimentés par des barrières physiques. Nous avons examiné la variation du mélanisme en fonction de l'urbanisation, des conditions environnementales locales (p. ex., la température et le couvert forestier), ainsi que de la structure géographique.
4. Contrairement aux tendances observées dans de nombreuses villes où le mélanisme atteint son niveau le plus élevé dans les centres urbains, le mélanisme à Montréal était généralement corrélé négativement avec l'urbanisation; toutefois, l'intensité et la direction des clines urbain‐rural variaient fortement à travers le complexe paysage insulaire de la ville. Des changements abrupts dans la fréquence des morphes coïncidaient avec les principaux cours d'eau, notamment le fleuve Saint‐Laurent et ses affluents, ce qui suggère que les rivières pourraient agir comme des barrières partielles à la dispersion, divisant le paysage en régions phénotypiques distinctes.
5. Conformément aux avantages thermorégulateurs du mélanisme, la fréquence du morphe mélanique augmentait davantage dans les zones caractérisées par des températures hivernales plus fraîches et un couvert forestier plus dense. La modélisation par équations structurelles a révélé que l'urbanisation supprime indirectement le mélanisme en modifiant ces deux variables.
6. Nos résultats suggèrent que les patrons phénotypiques au sein des villes ne sont pas uniformes, mais sont plutôt façonnés par une hétérogénéité environnementale à fine échelle et par des barrières géographiques. Ces résultats soulignent la nécessité de dépasser les simples contrastes urbain‐rural pour prédire la variation phénotypique au sein des populations urbaines.

## 1 INTRODUCTION

Anthropogenic activity is driving some of the fastest rates of evolution worldwide (Alberti, 2015; Hendry & Kinnison, 1999; Palumbi, 2001; Thompson et al., 2022). Urban environments impose novel selective pressures and generate convergent ecological conditions across the globe—including increased impervious surface cover, elevated temperatures, pollution, artificial lighting at night and fragmented green spaces—which in turn contribute to ecological homogenization (Caizergues et al., 2024; Groffman et al., 2014; McKinney, 2006). Because these features are widespread and replicated among cities, and because urban environments are the fastest‐growing ecosystem on Earth (United Nations, 2018), urban landscapes can function as compelling natural laboratories to test ecological and evolutionary hypotheses (Alberti et al., 2017; Johnson & Munshi‐South, 2017; Rivkin et al., 2019). Parallel evolution (i.e. the independent emergence of similar traits in separate populations exposed to comparable selective pressures; Bolnick et al., 2018; Futuyma & Kirkpatrick, 2017) is increasingly documented in urban environments, and urban–rural clines in phenotypic traits are often interpreted as evidence of adaptive evolution (Linnen & Hoekstra, 2010). For example, animal studies have shown that urban environments consistently select for behavioural traits, such as increased boldness and problem‐solving (Ducatez et al., 2017; Lowry et al., 2013; Sol et al., 2013), physiological traits, such as altered stress physiology and enhanced pollution tolerance (Partecke et al., 2006; Salmón et al., 2018), and morphological traits, such as reduced body size or changes in coloration (Alberti et al., 2017; Caizergues et al., 2022; Smith et al., 2021).

In spite of cities' highly convergent features, substantial variation in structure, land‐use history and climate factors can influence the predictability of evolutionary responses across cities and species (Fidino et al., 2021; Rivkin et al., 2019; Santangelo et al., 2022; Schmidt et al., 2020; Thompson et al., 2016). For example, Fidino et al. (2021) found that responses to urbanization in several species of mammals changed in magnitude and direction between cities depending on the proportion of green space and housing density, and Santangelo et al. (2022) demonstrated that urban–rural phenotypic clines can be spatially variable and context‐dependent across cities. In genetic studies of urban evolution, non‐adaptive mechanisms such as limited gene flow and increased genetic drift have been shown to act as key drivers of population divergence, given that urbanization often limits dispersal by fragmenting habitats and increasing landscape resistance (Miles et al., 2019; Rivkin et al., 2019). Together, these non‐adaptive processes combined with the potential for opposing selective pressures can generate complex or spatially variable phenotypic clines between cities (Santangelo et al., 2022).

In addition to variation among cities, substantial spatial and structural variation often exists *within* a single city. Natural and anthropogenic features, such as rivers, highways and dense development can act as semi‐permeable or absolute barriers within a city which can work to shape local population structure, as observed in species such as the red fox (*Vulpes vulpes*; Wandeler et al., 2003), northern dusky salamander (*Desmognathus fuscus*; Fusco et al., 2021; Munshi‐South et al., 2013) and white‐footed mouse (*Peromyscus leucopus*; Harris et al., 2016; Munshi‐South & Kharchenko, 2010). Furthermore, fine‐scale variation in environmental features, such as canopy cover, winter temperature, and patch connectivity, may generate localized phenotypic clines that diverge from general city‐wide patterns. Collectively, the interactions between landscape heterogeneity and restricted dispersal highlight the need to identify the mechanisms that might predict phenotypic and genetic distributions across multiple, nested spatial scales. Nevertheless, many empirical studies rely on coarse urbanization metrics or cross‐city comparisons which overlook fine‐scale spatial and ecological heterogeneity that can structure these patterns (Rivkin et al., 2019; Schell, 2018; Vasemägi, 2006). Addressing this gap will require a study design that explicitly incorporates landscape heterogeneity, potential barriers to gene flow, and environmental gradients. Our study leverages this framework by examining the eastern grey squirrel (*Sciurus carolinensis*) in Montreal, Canada, where major rivers, sprawling suburban development and variation in local microhabitat features—such as temperature, canopy cover, impervious surfaces and park and building area—provide an opportunity to investigate how fine‐scale landscape structure and local environmental conditions jointly influence coat colour morph distributions.

The eastern grey squirrel is one of the most abundant mammals living in urban environments in eastern North America (Gibbs et al., 2019; Merrick et al., 2016). Compared to other urban fauna, tree squirrels (*Sciurus* spp.) are highly capable of integrating into the urban matrix given their efficient habitat usage and resource exploitation (Mccleery et al., 2008; Parker & Nilon, 2008). Although grey is the standard coat colour morph for the eastern grey squirrel, a second common melanic morph exists as a result of a specific deletion in the Mc1R gene (McRobie et al., 2009). The melanic morph has become rare in recent centuries, a decline often attributed to widespread deforestation in North America (Allen, 1943; Schorger, 1949). Reduced crypsis in open‐canopy, new‐growth forests likely increased predation risk and mortality for melanics relative to grey morphs, which remain well‐camouflaged in second‐growth habitats (Benson, 2013; Bowers & Breland, 1996; McCleery et al., 2008), and the detectability of colour morphs also appears to shift with background substrate (Proctor et al., 2025).

Interestingly, the melanic morph continues to dominate in northern parts of the species' range, where increased thermoregulation might provide an adaptive advantage (Ciurej et al., 2019; Ducharme et al., 1989). Urban environments also frequently support stable populations of the melanic morph, and multiple mechanisms have been proposed to explain the prevalence of melanism in urban environments, including the sequestration of heavy metal pollutants within melanin granules (Goiran et al., 2017), enhanced immune response linked to melanocortin pleiotropy (Chatelain et al., 2016), increased hair toughness providing protection in dense urban habitats (Bonser, 1995), improved visibility on paved surfaces reducing road mortality (Parlin et al., 2025), and relaxed selective pressure against melanism under modified predator regimes where non‐natural mortality factors predominate (Bonar et al., 2025; Cosentino et al., 2023; McCleery et al., 2008).

Recent studies have documented variation between cities in the strength of urban–rural clines in eastern grey squirrel melanism, with the steepest clines occurring in densely populated and sprawling urban areas with colder temperatures and high forest cover (Cosentino & Gibbs, 2022). While these patterns are consistent with isolation‐by‐distance at broad scales, finer within‐city analyses are required to determine whether similar clines arise from fine‐scale spatial and environmental gradients. Our study addresses this gap in knowledge by conducting a detailed, spatially explicit investigation of colour morph distribution within a single metropolitan area, where sprawling suburban development is intersected by several major rivers that might act as barriers to gene flow.

Specifically, we examined patterns of melanism in eastern grey squirrels across the Montreal Metropolitan Area (*Sciurus carolinensis*) using a combination of visual surveys, SquirrelMapper data (Cosentino & Gibbs, 2022), and citizen science observations from iNaturalist. Montreal's central island core is intersected by major rivers that separate it from the surrounding suburban areas, creating a fragmented and highly heterogeneous landscape (Dupras et al., 2016). This setting allowed us to investigate how both fine‐scale environmental variation (e.g. winter temperature, canopy cover, park size, impervious surfaces and surrounding building area) and geographic structure influenced coat colour morph distributions. We asked: (1) whether urban–rural patterns in melanism were consistent across directional/spatial transects, (2) which environmental variables best explained spatial variation in melanism and (3) how geographic features, such as rivers and regional subdivisions, affected morph distributions across the city. We hypothesized that urban–rural patterns in melanism would vary across transects and would be mediated by local environmental heterogeneity. Specifically, we predicted that urbanization would influence melanism through three primary routes: (1) a direct pathway representing novel anthropogenic pressures not captured by environmental proxies, (2) a thermal pathway, where urban heat island effects relax selection for thermoregulation and (3) a habitat pathway, where high‐intensity urban areas are characterized by fragmented habitats, reduced park area, and lower canopy cover, diminishing microenvironmental buffering and altering selection on camouflage and microhabitat usage. Lastly, we hypothesized that geographic barriers would act as a significant confounder, producing localized shifts in melanism independent of the immediate urban gradient.

## 2 MATERIALS AND METHODS

Our study is correlative in nature and cannot conclusively establish causation. Nonetheless, correlations are a critical first step in identifying which causal hypotheses merit further investigation, and we treat them as such throughout. We therefore discuss which mechanisms could plausibly underlie the associations we observe, while acknowledging that confirmation will require experimental manipulation or targeted hypothesis testing. In this spirit, we use our structural equation model, specified a priori from our hypothesized causal structure and represented graphically in Figure 4, as our primary framework for evaluating whether the pattern of associations is consistent with specific indirect pathways.

### 2.1 Study area

**FIGURE 1:**
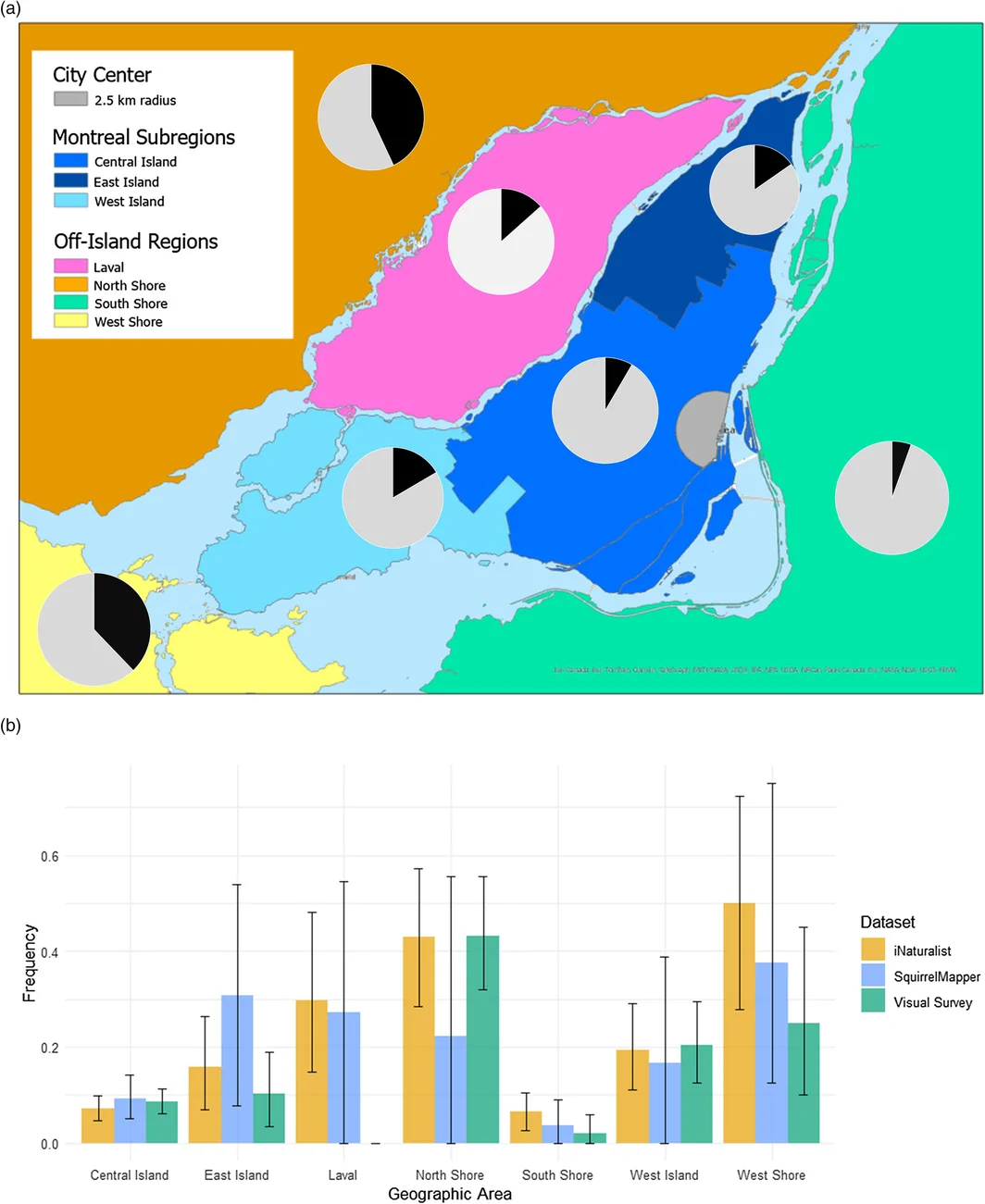
(a) Boundaries of the major regions used in our study and proportion of melanic squirrels in the pooled data frame. The proportion of melanic individuals in each major region (pooled dataset) is indicated by the black area of each pie chart. (b) Proportion of melanic squirrels in each area by dataset. The North and West Shores had the highest melanism prevalence (~40%) whereas the South Shore had the lowest (~6%). Peripheral islands are associated with their municipality of administration. Error bars represent 95% Wilson score confidence intervals.

**FIGURE 2:**
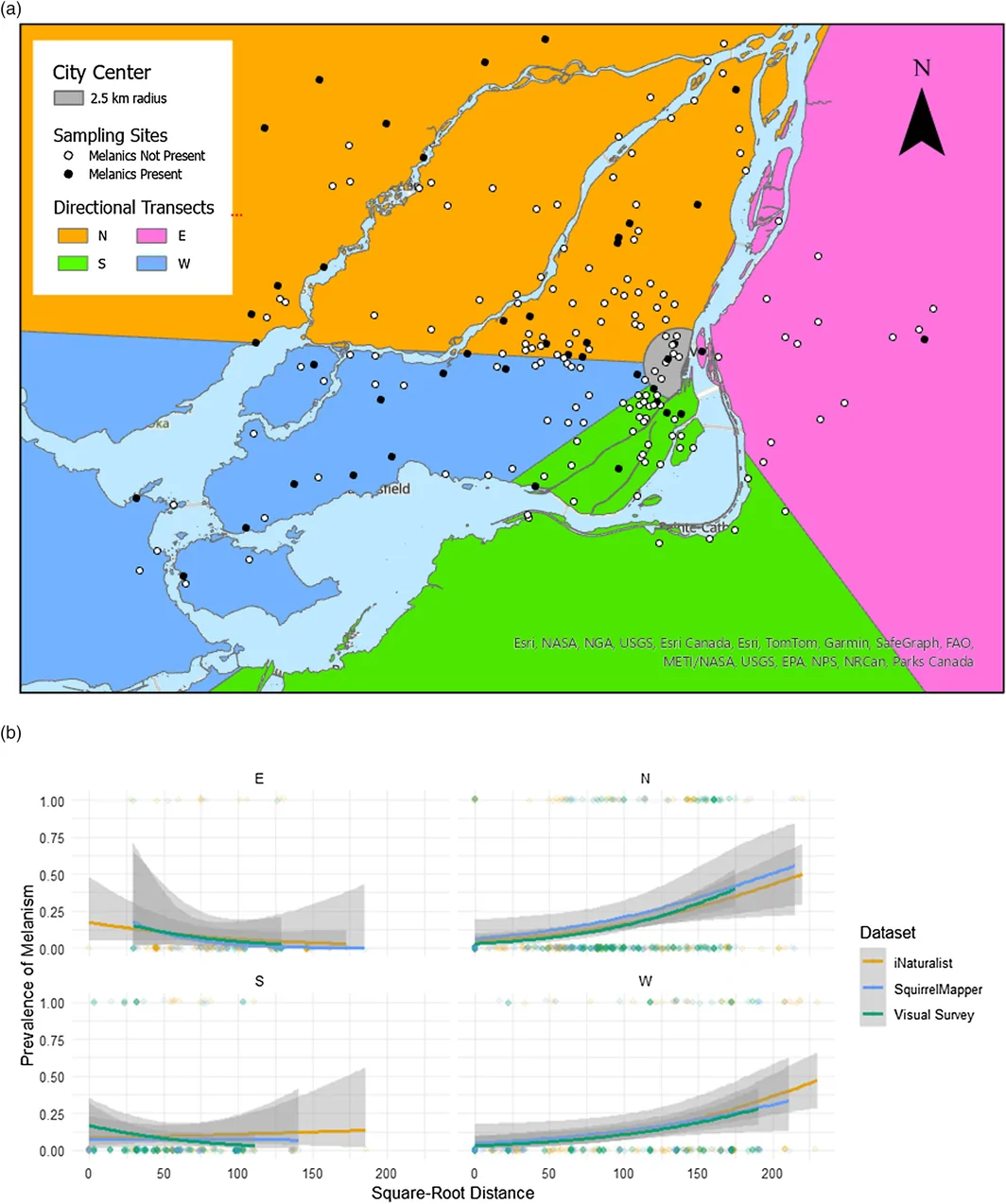
(a) Map of the study area divided by the four directional transects. Points on the map mark our visual survey sampling sites. Black points indicate the presence of melanic individuals while white point indicates their absence. (b) Prevalence of Melanism by Distance from City Center. Directional trends are similar across all three datasets.

We defined the Montreal Metropolitan Area as the intersection between the Montreal Census Metropolitan Area (Statistics Canada, 2023), and the Montreal Metropolitan Community (Communauté Métropolitaine de Montréal, n.d.) to ensure that the study area captured the regions recognized by both federal and municipal authorities. Within this boundary, we identified five regions physically separated by major rivers: (1) the Island of Montreal, (2) Laval, (3) the South Shore, (4) the North Shore, and (5) the West Shore which were used for regional analysis. We further separated the Island of Montreal into the West, Central and East Island (Figure 1) following media and municipal conventions (The Canadian Encyclopedia, n.d.; Ville de Montréal, 2021). These subregions are often used to describe land‐use differences within the Island of Montreal, but because these boundaries lack formal geographic delimitation, we treated them as descriptive groupings for data visualization rather than ecologically distinct units. We designated the city centre as a circular area with a 2.5 km radius centred on the coordinates for the geographic centroid of the urban core, as defined by Natural Resources Canada (45°30′32″ N, 73°33′15″ W). We then established 4 directional transects broadly following cardinal directions, adjusted to minimize river crossings (Figure 2). This ensured each transect spanned a relatively continuous landmass and avoided introducing discontinuities caused by major water barriers.

### 2.2 Eastern grey squirrel observations

We classified all squirrels as either melanic (black to dark‐brown pelage) or non‐melanic, applying this criterion consistently across all three data sources described below. We combined observations from all three sources into a single dataset, retaining geographic coordinates, morph identity, date and source identifier for each record. These sources are: (Adams et al., 2004) visual field surveys (Alberti et al., 2017) citizen science observations from iNaturalist (iNaturalist, 2024), and (Alberti, 2015) the SquirrelMapper dataset (Cosentino & Gibbs, 2022). This study did not require licences, permits, or ethical approval as data collection consisted solely of non‐invasive observational surveys and analysis of publicly available citizen science photographs.

#### 2.2.1 Visual survey dataset

We created the Visual Survey Dataset by conducting visual surveys in 197 parks across the Montreal Metropolitan Area, in the months of June and July of 2022, between 10:00 and 16:00, to minimize biases in squirrel activity and detectability (Engel et al., 2020; Larson & Sander, 2022; Thompson, 1977). At each site, we conducted a 20‐min survey, during which we recorded the coat colour of all visible eastern grey squirrels. Starting with the first squirrel encounter, we walked along designated park trails without backtracking or retracing our steps. If we did not see any squirrels after 20 min of continuous searching, we recorded the site as empty.

#### 2.2.2 iNaturalist dataset

We constructed the iNaturalist dataset by compiling all sightings denoted as ‘Research‐Grade’ for *S. carolinensis* that were uploaded to the iNaturalist database (iNaturalist, 2024) between the months of May to August in 2021, 2022 and 2023. We only used sightings with attached photos or videos, which allowed for visual inspection and classification of the squirrels' coat colour. The method for morph assignment was identical to that used for the Visual Survey dataset.

#### 2.2.3 SquirrelMapper dataset

We assembled the SquirrelMapper dataset using data that is publicly available in an online repository (Cosentino & Gibbs, 2022). This dataset combines several sampling methods, including citizen science reporting, visual surveys and camera traps with pre‐assigned morphs' identities. We restricted our dataset to observations between the months of May and August to match the seasonal window of the other two datasets. Observations ranged from 28 May 2003 to 20 October 2020.

### 2.3 Spatial data

We conducted all spatial analyses in R 4.5.0 (R Core Team, 2024). We computed distance from the city centre polygon—our initial proxy for urban intensity (Szulkin et al., 2020)—for each of the points in the combined dataframe using the st_distance() function from the sf package (Pebesma, 2018). For each park sampled in the Visual Survey Dataset, we extracted a suite of environmental data, including monthly surface temperature, canopy cover, impervious cover, park area, surrounding building area, surrounding road area and surrounding building height, to assess microhabitat influences on colour morph distribution. These variables collectively represent the primary axes of urban environmental variation that have been shown to influence wildlife ecology and phenotypic evolution in urban systems. Specifically, land surface temperature is used to infer thermoregulatory selection pressure. Canopy cover reflects vegetation structure which is relevant to both microclimate buffering and crypsis‐based selection. Impervious surface cover, surrounding building area, building height and road area together capture complementary dimensions of urban intensity and structural habitat modification. Lastly, park area is a measure of habitat integrity, with larger parks more likely to maintain stable environmental conditions or microclimates that are often disrupted in smaller, more exposed areas (Groffman et al., 2014; McDonnell & Hahs, 2008; Szulkin et al., 2020).

We calculated park surface area using the City of Montreal's park polygon dataset. For parks located outside the island of Montreal, we extracted boundaries using the osmdata package (Padgham et al., 2017) with OpenStreetMap data. We calculated polygon areas using the st_area() function from the sf package (Pebesma, 2018). To quantify canopy and impervious surface cover, we used the Metropolitan Canopy Index dataset (Communauté Métropolitaine de Montréal, 2023). We intersected park boundaries with this raster and calculated the proportion of pixels classified as canopy or impervious.

To characterize the surrounding built environment, we created a 100 m buffer around each park. We extracted building counts and building footprint areas from Microsoft's Canadian Building Footprints dataset (Microsoft Corporation, 2019) and calculated the proportional building area by dividing the total building footprint area by the buffer area. We estimated average building height by extracting elevation values at each building centroid from the Canadian High Resolution Digital Elevation Model (Natural Resources Canada, 2017).

To estimate proximity to major infrastructure, we defined major roads as Trans‐Canada highways, national highway system segments and other major and secondary roads. We used the National Road Network dataset (Statistics Canada, 2024) and computed the distance from each park to the nearest major road using the st_nearest_feature() function from the sf package (Pebesma, 2018). We calculated total road surface area within 100 m buffers using the City of Montreal's roadway asset dataset (Ville de Montréal, 2009), which includes roads, intersections, alleyways, and sidewalks. We restricted these calculations to parks on the Island of Montreal due to data availability.

We estimated land surface temperature for each park using Landsat 8 satellite imagery and a published open‐source workflow (Ermida et al., 2020). We computed mean land surface temperature values for each month of 2022. We performed both land surface temperature and building height calculations using Google Earth Engine (Gorelick et al., 2017).

### 2.4 Statistical analysis

We conducted all statistical analyses in R 4.5.0 (R Core Team, 2024). Before modelling, we tested whether melanism showed spatial clustering across the combined dataset using Moran's *I* (Bivand & Wong, 2018). We coded morph identity as a binary variable (melanic = 1, non‐melanic = 0) and constructed an inverse distance weight matrix from each observation's geographic coordinates. Records with identical coordinates received a small non‐zero distance offset (1 × 10^−8^ decimal degrees) to avoid division‐by‐zero errors.

To estimate the primary spatial axis of melanism variation across the city, we fit a generalized linear model (GLM) on the combined dataset using the glm() function, with morph identity as the binary response variable and both latitude and longitude as fixed effects. We used the coefficients from this binomial GLM (latitude and longitude) to define a spatial gradient vector, where the direction of maximum increase was calculated as the arc‐tangent of the ratio of these coefficients. To account for potential variation between datasets, we initially included dataset as a random effect in a generalized linear mixed model (GLMM) using the glmer() function from lme4 package (Bates et al., 2015). However, because this grouping variable contained only three levels, the random effect resulted in a singular fit. Following the recommendations of Oberpriller et al. (2022), who demonstrate that models with singular fits for random effects can be unreliable, we opted to drop this grouping variable to ensure parameter stability and chose to fit a GLM, further supported by the significant overlap in confidence intervals (Figures 1 and 2).

Using a separate GLM, we examined the extent to which the urban–rural divergence in melanism was consistent across directional transects. We modelled transect direction, distance from the city centre, and their interaction as fixed effects to test whether the strength of the urban–rural cline differed among directions. We then fit separate logistic regression models for each geographic region (i.e. the Island of Montreal, Laval, the South Shore, the North Shore and the West Shore) to examine how the urban–rural melanism relationship varied across areas within the city, independent of transect direction. This approach allowed us to identify region‐specific clines without the spatial confoundings introduced by a global interaction term.

To explore the effect of environmental variables in the melanism‐urbanization cline, we first explored the environmental data to assess variable distributions and determine which predictors were appropriate for analysis. We used only the Visual Survey Dataset for this analysis because each sampling site represented an independent population surveyed with a consistent, standardized protocol. Limiting the analysis to this dataset allowed us to avoid potential biases arising from differences in sampling design and data quality among datasets. We conducted all visual surveys within a distinct city park, and we used the park boundaries to determine the area for which we extracted microhabitat environmental variables. This removed the need to create buffers around each individual observation. We constructed a correlation plot using the corrplot package (Wei & Simko, 2024) to screen for collinearity. We chose to exclude summer temperature because it was highly correlated with common urbanization proxies (distance from city centre, % impervious cover, % surrounding building area, mean building height). Furthermore, because mortality, and therefore selection, is most likely to occur during the winter, when cold stress and food scarcity are greatest and squirrels are most likely to exploit anthropogenic food sources (Adams et al., 2004; Rimbach et al., 2023), we retained temperature during the coldest month of winter (January) as our thermal variable. To extract a single measure for urbanization among these proxies, we conducted a Principal Component Analysis (PCA) on our Visual Survey dataset. From the missMDA package (Josse & Husson, 2016), we used the imputePCA() function to impute missing data and used the prcomp() function to run the PCA. We then extracted PC1 to calculate an Urbanization Index for each park. We used this Urbanization Index to capitalize on urbanization as a complex multivariate metric which may affect squirrel ecology in a variety of latent ways.

To assess the individual and interactive contributions of the environmental variables specified in our a priori causal structure, we used an exploratory model selection procedure across the same candidate predictors plus their pairwise interactions. We fit a global binomial GLM with the melanism ratio per park as the response variable (weighted by squirrel counts) and Urbanization Index, January temperature, canopy cover, park area, and all pairwise interactions as predictors. We then performed all‐subsets model selection starting from this full‐factorial model using the dredge() function in the MuMIn package (Bartoń, 2025), which enumerates all candidate models that are subsets of the specified global formula. We ranked candidate models by AICc and extracted summaries for the most strongly supported models (within 2 AICC units of the top model) to inspect fit and coefficient patterns. Finally, we computed summed Akaike weights for each term using the sw() function from package MuMIn (Bartoń, 2025) to quantify variable importance across the candidate set and model.avg.() to average effects across the top models. Then, to test whether geographic structure explained residual variation beyond our environmental model, we regressed the residual variation in the global environmental GLM against either Direction or Region. Significant associations would indicate that geographic features produce morph frequency shifts independent of the local environmental gradient.

Next, we implemented a structural equation model (SEM) using the sem() function in the lavaan package (Rosseel, 2012), to assess whether the hypothesized relationships between urbanization, environmental variables, geography, and melanism were consistent within a unified framework. We used the robust maximum likelihood estimator (MLR) with full information maximum likelihood (FIML) to account for missing data. We specified all pathways a priori based on our hypothesized causal structure (Figure 4) and retained them regardless of significance, treating non‐significant paths as informative results rather than grounds for removal. Where the initial global model showed poor fit, we applied targeted structural refinements, such as freeing correlated residuals until acceptable fit was achieved (CFI >0.95, RMSEA <0.08, SMR <0.08). We did not pursue further simplification beyond this point, as removing hypothesized paths would constitute data‐driven model modification rather than hypothesis testing. Refinement decisions were guided by ΔBIC >10 followed by Satorra‐Bentler scaled chi‐square difference tests (*p* > 0.05). This approach allowed us to model direct and indirect effects simultaneously, account for latent covariance among predictors, and test the overall fit of the causal structure to the data.

## 3 RESULTS

We observed melanic squirrels in 49 of the 197 parks sampled for the Visual Survey dataset, and melanic squirrels represented 12.8% of the 1734 squirrel observations across the combined dataset (Visual Survey: 13.1%, *n* = 750; iNaturalist:12.9%, *n* = 730; SquirrelMapper: 11. 8%, *n* = 254; Figure 1 and Figure S1). No squirrels were detected in 52 of the 197 parks, which we marked as empty (Figure 2). The Central Island subregion contributed the largest number of observations (Figure S1). Melanism showed modest but highly significant spatial autocorrelation (Moran's *I* = 0.071, *p* = 7.27 × 10e‐10), indicating that morph frequencies are spatially structured across the city, though the effect size suggests this clustering is relatively weak and does not preclude the use of standard regression approaches. The random intercept for dataset was estimated at zero, indicating negligible among‐source variation in baseline melanism frequency. We therefore removed it from downstream analyses.

Melanism was not uniformly distributed across the city. We observed low frequency near the city centre (~5%) and higher frequency as distance from the city centre increased (Figure 1). However, this pattern was primarily driven by high levels of melanism in rural regions in the north and west (~58% and 37% of individuals). Melanism was low in suburban and rural regions in the south and east (~5%), indicating no cline in melanism, or even a weak decrease in melanism, in those directions (Figure 2). The directional transect GLM confirmed that melanism increased significantly with latitude (*β* = 0.4285, SE = 0.065, *p* = 5.87e‐11) and decreased with longitude (*β* = −0.6186, SE = 0.078, *p* = 1.65e‐15), indicating a northwest‐directed increase in melanism (~305° azimuth). In our model quantifying the interaction between urbanization and transect direction, melanism significantly decreased with distance from the city center (*β* = −0.014, SE = 0.0071, *p* = 0.044), but this effect was modulated by direction: the interaction terms for the north (0.0292, SE = 0.0075, *p* = 1.07e‐4) and west transects (0.0277, SE = 0.0076, *p* = 2.66e‐4) showed positive distance effects, consistent with the northwest directional signal, while south and east transects did not.

**FIGURE 3:**
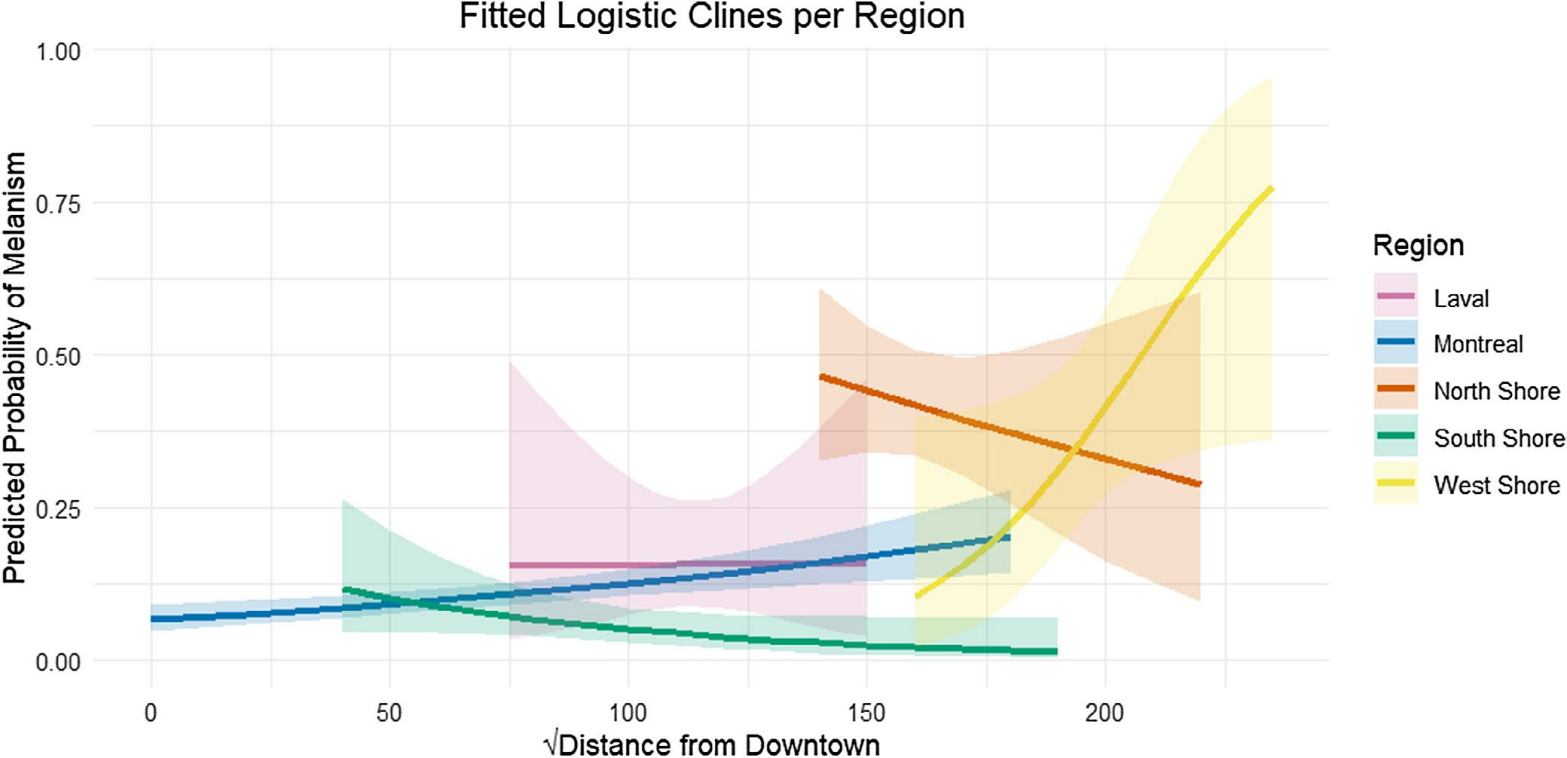
Distance‐Melanism clines for each of the major regions. All three datasets were pooled (i.e. Visual Survey, iNaturalist, SquirreMapper).

While the directional model established a clear northwest‐oriented cline, analysis by geographic region revealed significant spatial heterogeneity (Figure 3). We observed that the overarching directional trends were driven by distinct regional clusters and localized clines rather than a uniform gradient. On the West Shore, melanic frequency rose significantly with distance (*β* = 0.046, SE = 0.023, *p* = 0.045). Melanism also weakly but significantly increased with distance on the Island of Montreal (*β* = 0.007, *p* = 2.01e‐4). In contrast, distance had a negative effect on melanism in the South Shore (*β* = −0.015, SE = 0.00841, *p* = 0.069) while having a non‐significant effect on Laval (*p* = 0.991) and the North Shore (*p* = 0.386). These results indicate that the macro‐scale directional trend is anchored in localized changes in melanism in different regions, rather than a uniform effect of distance from the urban core.

Next, we identified the role of various environmental variables on melanism, as well as their interaction with urbanization. We found that distance, impervious cover, building height and building area were highly correlated along a single dimension (Figure S2), supporting our choice to conduct dimensionality reduction on these variables. After running a PCA, we found PC1 to represent 49% of variance, followed by PC2 at 24%, and PC3 at 16%. Based on factor loadings, we chose PC1 as a direct measure of urban intensity, with a negative loading for distance from city centre and a positive loading for impervious cover, building height, building area, and road area. Based on ecological relevance and diagnostic screening for multicollinearity, we retained canopy cover, winter temperature, urbanization and park size as the primary candidate variables for model selection. Across all study sites, environmental gradients were substantial: average winter temperature varied from −8.967 to −2.306°C, canopy cover varied from 1% to 97.6%, park area varied from 1353 to 9,133,699 m^2^ and urbanization varied from −4.07 to 5.09.

AICc‐based model selection across the candidate set identified canopy cover (sw = 0.96) and winter temperature (sw = 0.95) as contributing most consistently to model fits across the candidate model set, while urbanization (sw = 0.61) and park area (sw = 0.55) appeared less consistently (Figure S3). Canopy cover and winter temperature appear in all top models (4 models within 2 AICc units) with the best‐supported model including only those two predictors (Table S1). Averaging across the top models, higher canopy cover was positively associated with melanism (*β* = 1.31, SE = 0.46, *p* = 0.00430), while warmer winter temperature was negatively associated with melanism (*β* = −0.53, SE = 0.18, *p* = 0.00385). Critically, the weak and inconsistent support for the Urbanization Index aligns with our hypothesis that urbanization does not directly predict melanism once local vegetation structure and winter microclimate are accounted for, but instead influences melanism indirectly through these environmental mediators. After accounting for environmental predictors in the GLM, we examined whether the residual variation in our model showed variation by geographic transect. We found no significant relationship between residuals and directional transects (*F* = 0.3954, *p* = 0.507). Instead, we found a strong relationship between residuals and region (multiple *R* ^2^ = 0.18, *F* = 7.9214, *p* = 8.733e‐06), showing that, after accounting for environmental effects, heterogeneity was shaped by regional differences rather than unified directional environmental gradients.

To further explore whether the observed associations among urbanization, environmental variables, geographic structure and melanism were mutually consistent, we constructed a hypothesis‐guided piecewise structural equation model (SEM). Sequential model comparison supported removal of all park area paths (ΔBIC = 365.83), consistent with the weak support for park area in our GLM model selection. We subsequently removed urbanization → melanism (ΔBIC = 3.1, Δ*χ* ^2^ = 0.80, *p* = 0.370) and region → winter temperature (ΔBIC = 3.1, Δ*χ* ^2^ = 2.31, *p* = 0.129). Removal of canopy → melanism was not supported (ΔBIC = 0.7, Δ*χ* ^2^ = 3.71, *p* = 0.054; AIC increase), so this path was retained in the final model despite a non‐significant coefficient, as its removal worsened fit.

**FIGURE 4:**
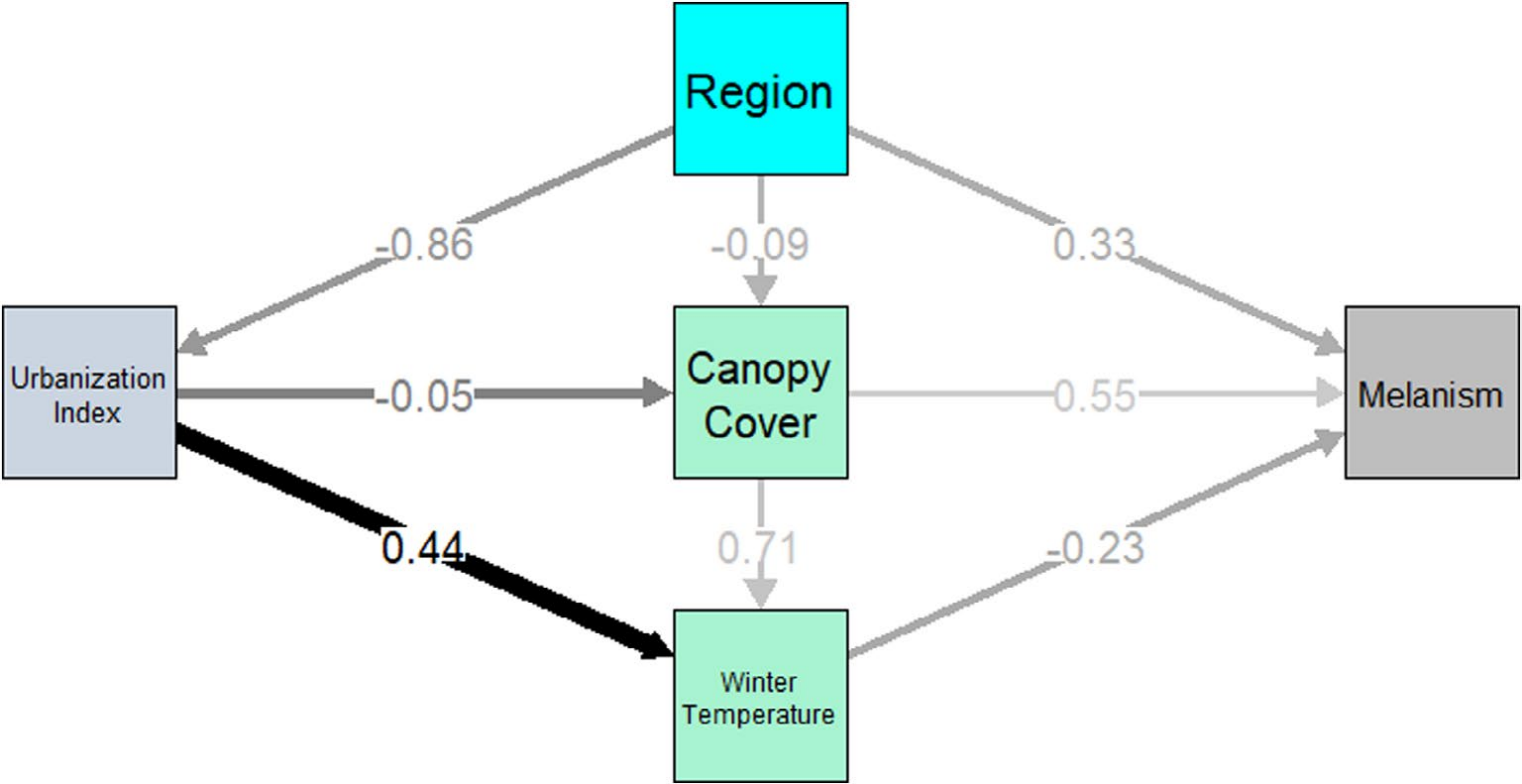
Pathways of our structural equation model. Pathway coefficients are shown on the arrows; pathway significance is represented by the thickness and darkness of the arrows.

The final model showed excellent fit (Yuan‐Bentler scaled *χ* ^2^ = 2.39, df = 2, *p* = 0.302; CFI = 0.999; RMSEA = 0.025; SRMR = 0.022) and explained 9.8% of variance in urbanization, 14.5% in canopy cover, 55.8% in winter temperature and 18.6% in melanism (Figure 4). Urbanization strongly predicted both environmental mediators: higher urbanization predicted reduced canopy cover (*β* = −0.385, *p* = 1.30e‐3) and warmer winter temperatures (*β* = 0.784, *p* < 1e‐15). In turn, greater canopy cover increased melanism (*β* = 0.159, *p* = 1.58e‐1) and warmer winter temperatures reduced melanism (*β* = −0.262, *p* = 1.01e‐2). The absence of a direct urbanization → melanism path, combined with supported indirect paths through both environmental mediators, is consistent with our hypothesis that urbanization acts on melanism through its effects on local microhabitat and microclimate. Regions were associated with differences in urbanization (*β* = −0.313, *p* = 2.47e‐7), canopy cover (*β* = −0.226, *p* = 2.8e‐2), and baseline melanism frequency (*β* = −0.246, *p* = 7.41e‐5). These effects indicate that baseline differences in phenotype frequencies remain across regions even after accounting for urbanization, canopy cover and winter temperature. These results suggest that environmental covariates and geographic structure jointly contribute to spatial variation in melanism. The consistency between our exploratory GLM and hypothesis‐guided SEM strengthens confidence across these findings.

## 4 DISCUSSION

Urban environments provide opportunities for replicated natural experiments which can help shed light on the ways in which ecological and evolutionary processes respond to human‐altered landscapes. Although past studies on urban evolution have primarily focused on broad urban–rural contrasts across cities, our results demonstrate that phenotypic patterns can also be highly heterogeneous within cities. We found that melanism in eastern grey squirrels did not follow a simple, uniform urban–rural cline in the Montreal Metropolitan Area, with morph frequencies varying markedly among regions and directional transects, revealing a spatial mosaic influenced by the physical barriers that fragment the city's landscape. Canopy cover and winter temperature also emerged as significant predictors of melanism, with urbanization working to mediate these predictors without directly influencing morph presence. Our findings highlight that patterns of phenotypic distribution in urban environments can emerge from the interaction between environmental gradients and geographic structure at fine spatial scales.

Specifically, we found that eastern grey squirrel melanism increased most towards the northwest, perpendicular to the Saint‐Lawrence River, with high frequencies in northern and western regions and low frequencies in southern and eastern areas. This directional melanism gradient likely reflects two converging influences: (1) the regional proximity of Montreal to the Great Lakes basin which lies to the west and serves as a major reservoir for the melanic morph (Lehtinen et al., 2020) and (2) the transition into the colder, more contiguous northern forests reflecting the broader latitudinal gradient documented across the species' range (Cosentino & Gibbs, 2022). The latter may be particularly relevant given that rural forests in northern and remote areas of Canada remain relatively undisturbed by human activity (Statistics Canada, 2018). The pattern we observed mirrors a recent continent‐wide study on eastern grey squirrel genetic structure showing that the Ontario‐Great lakes genetic cluster, where melanism is most prevalent, contrasts with the East Coast genetic cluster, where melanism is extremely rare (Fusco et al., 2024).

We also found heterogeneity in phenotype clines that aligned with the major rivers that segment the Montreal Metropolitan Area. These rivers might limit dispersal, facilitate non‐adaptive divergence by restricting gene flow, and contribute to spatial discontinuities. In particular, the Saint‐Lawrence system could mark the approximate biogeographic boundary between the Ontario‐Great lakes genetic cluster and the East Coast genetic cluster, such that squirrels on either shore exhibit closer genomic affinity to one cluster or the other. This inference is supported not only by the sharp melanism transitions we observed across regional boundaries, but also by analogous patterns of genetic clustering across the Saint‐Lawrence in other taxa, including white‐footed mice (*Peromyscus leucopus*; Fiset et al., 2015) and common raccoons (*Procyon lotor*; Cullingham et al., 2009). Nevertheless, expanding the geographic reach of our visual surveys through each directional transect would help define the full spatial extent of the melanism gradient. Complementarily genomic sampling across all five regions of our study area, particularly on opposite sides of riverbanks, is essential to rigorously test the Saint Lawrence's role and strength as a barrier, as current inferences for Montreal are based on only nine individuals sampled near the urban core, all from the Island of Montreal (Fusco et al., 2024).

Our environmental analyses identified canopy cover and winter temperature as proximate predictors of melanism, with urbanization operating exclusively through these mediators rather than directly. This indirect relationship is consistent with the urban heat island effect and vegetation loss in urban cores shaping the selective landscape for coat colour morphs (Groffman et al., 2014). Warmer winters in urban cores may relax thermoregulatory selection (Charmantier, 2024; Diamond et al., 2017) favouring the melanic morph, consistent with the persistence of melanism at high frequencies in the cold, forested rural areas to the north and west of the Montreal Metropolitan Area. Additionally, reduced canopy cover in urban areas might shift the balance of crypsis‐based selection against melanism, consistent with the historical decline of the melanic morph following widespread deforestation across North America (Allen, 1943; Benson, 2013). Together, these two indirect pathways suggest that urbanization can reshape the adaptive landscape for coat colour through its effects on microclimate and vegetation structure, rather than through any single direct selective force. Our results parallel the cline reversals in grey squirrel colour morph observed in Toronto and Kitchener, cities similarly located along the north bank of the Great Lakes–Saint‐Lawrence basin (Cosentino & Gibbs, 2022). In these high‐latitude systems, the surrounding cold and extensively forested rural landscapes emulate pre‐urban forest conditions that historically favoured melanism. The prevalence of melanism in Montreal's northern and western rural sectors likely reflects the persistence of this regional adaptation, where the thermal and structural selective environment continues to permeate the landscape, favouring the melanic morph outside the city and eclipsing urban‐specific advantages.

Despite its strengths, our study has several important limitations. First, the data used in our study represents a phenotypic snapshot and cannot definitively demonstrate that evolution is occurring. Distinguishing adaptive evolution from demographic stochasticity or historically structured variation requires either direct fitness measurements linking coat colour to survival and reproduction, or temporal data spanning multiple generations. The approximately 20‐year window of this dataset is insufficient to distinguish a stable evolutionary signal from fluctuating selection, particularly given the rapid pace of urban change. Our findings are therefore best interpreted as evidence of selective forces that, if persistent across generations, could drive evolutionary divergence. Second, our visual surveys focused only on parks, potentially overlooking morph variation in other urban habitats, such as residential neighbourhoods, commercial areas and street corridors, where squirrels are also abundant. If melanism frequencies differ between park and non‐park urban habitats, as might be expected if pavement background matching or road mortality impose differential selection outside of green spaces, our SEM estimating urban melanism frequency could be biased. As such, future surveys should explicitly sample squirrels across the full range of urban habitat types. Third, although grey squirrel activity typically peaks in the morning and evening, our visual surveys were conducted between 10:00 and 16:00 to maximize consistency across sites. While we have no evidence that melanic and grey morphs differ in their midday activity, lower overall detection rates during this window may have reduced precision in site‐level frequency estimates. Fourth, morph‐specific detectability may introduce bias into our environmental association analyses. The visual contrast of grey and melanic morphs against their surroundings is highly background‐dependent; melanic individuals are notably more difficult to detect in complex or shaded environments, but easier to detect against the light‐coloured, impervious surfaces which are common in cities (Cosentino et al., 2023; Proctor et al., 2025). Citizen science datasets may also be subject to reporting bias if observers differentially record melanic individuals because they are perceived as more novel or striking than grey morphs. The broad congruence in morph frequency estimates across our three datasets suggests that this bias is not severe; nonetheless, we cannot exclude the possibility that melanism might be modestly overrepresented in the iNaturalist and SquirrelMapper records, relative to true population frequencies. If detectability differs systematically between morphs across environmental gradients associated with visual complexity (e.g. canopy cover or urbanization), our estimates of the association between these factors and melanism could be biased in ways that the current data cannot fully correct for. We note that this concern applies most acutely to fine‐scale environmental associations, rather than overarching regional patterns, which are unlikely to be reversed by detectability differences alone. Fifth, although we weighted our park‐level GLM by squirrel count, the low per park squirrel counts in our visual survey dataset might reduce the precision of our site‐level estimates of morph frequencies. We treated parks as sampling units and modelled proportions rather than raw frequencies to mitigate the influence of varying sample sizes. Nevertheless, results from parks with few counts should be interpreted cautiously. Finally, we did not implement protocols to explicitly prevent double‐counting of individuals within or across datasets. Repeat observations of the same individual remain possible and could inflate the effective sample sizes at frequently visited sites, though the large spatial and temporal scale of the combined dataset likely buffers aggregate estimates against the influence of any single individual (Hochachka et al., 2012).

These limitations define several key empirical gaps that future research could address. Beyond genomic sampling across regions, several additional approaches could strengthen the conclusions of this study. Population density data across the urban–rural gradient would help clarify whether the observed morph variation reflects demographic stochasticity or adaptive differentiation. Eastern grey squirrels often show higher urban occupancy (Anthonysamy et al., 2025), but the ecological consequences of urban–rural density differences for genetic structure remain poorly characterized (Hahs et al., 2023). Additionally, assessing how density shapes spatial genetic structure would help clarify whether the observed variation in melanism reflects drift or selection, and museum specimens could provide the temporal baseline needed to assess whether current morph distributions represent a stable historical pattern or a recent response to urban change. The 19th‐century Lachine Canal, which segments the southern portion of the Island of Montreal, presents a compelling opportunity to compare the barrier effects of natural versus anthropogenic waterways and to examine how the timeline of barrier establishment relates to the degree of phenotypic differentiation. Lastly, explicit modelling of landscape resistance, incorporating river width, flow velocity, seasonal ice cover and the location of anthropogenic crossing points, would provide a more mechanistic test of the dispersal barrier hypothesis than our regional analysis alone.

Overall, our results indicate that fine‐scale interactions between environmental gradients and geographic structure can influence the spatial distribution of phenotypes in urban environments. Our findings build upon a growing body of literature which demonstrates that phenotypic distributions in urban systems are closely linked to landscape structure and spatial heterogeneity across cities (Miles et al., 2021; Richardson et al., 2014; Rivkin et al., 2019; Szulkin et al., 2020). This study highlights the importance of explicitly incorporating fine‐scale within‐city spatial variation into urban evolutionary research. It offers a detailed characterization of morph frequency patterns within a single city and insights into how landscape and environmental features may drive phenotypic divergence.

## AUTHOR CONTRIBUTIONS

Francis Quang‐Hai Dinh designed the data collection methodology, collected squirrel observations and compiled online squirrel data. Isabella C. Richmond compiled environmental data using GIS methods and analyses. Francis Quang‐Hai Dinh performed statistical analyses and led the writing of the manuscript. Andrew P. Hendry and Allison M. Roth provided guidance and feedback on analysis and manuscript development. All authors contributed critically to the drafts and approved the final version for publication. This study was conceived, designed, and conducted by researchers all based in Montreal at the beginning of the study, with lived experience and direct familiarity with the study region. All stages, from research design and data collection to analysis and interpretation, were carried out locally, ensuring that regional ecological knowledge and context‐specific considerations were incorporated throughout. Literature from Canadian and Québec‐based researchers was cited wherever relevant to situate this work within the local research landscape.

## CONFLICT OF INTEREST STATEMENT

All authors declare that they have no conflicts of interest.

## Supporting information


**Figure S1.** Distribution of squirrel sightings across regions across Regions and Subregions and datasets.
**Figure S2.** Heat map to show autocorrelation between select variables. Negative correlations are in red while positive correlations are in blue.
**Figure S3.** Variable importance based on summed Akaike weights in our model dredge. Winter temperature and canopy cover are more important predictors of melanism than urbanization and park size. Interaction effects showed substantially lower importance weights compared to all primary environmental predictors.
**Table S1.** Top 10 GLM dredge models by AICc. Canopy cover and winter temperature had large effect sizes and were significant across all models. Four models (bold) are within 2 AICc units of the top model.

## Data Availability

All data and analysis code supporting the findings of this study is available from the Zenodo Digital Repository https://doi.org/10.5281/zenodo.20586852.
