# Supplementary material for: Fine‐scale spatial variation in eastern grey squirrel melanism across a complex urban landscape
Source: J Anim Ecol. 2026 Jul 22;95(9):1778–92. doi: 10.1111/1365-2656.70295 (PMC13536263; doi:10.1111/1365-2656.70295)
Supplement: Supplementary file 1 [file JANE-95-1778-s001.docx]

**Supplementary Materials**

Figure S1.

Distribution of squirrel sightings across regions across Regions and Subregions, and datasets.

Figure S2.

Heat map to show autocorrelation between select variables. Negative correlations are in red while positive correlations are in blue.

Figure S3.

Variable importance based on summed Akaike weights in our model dredge. Winter temperature and canopy cover are more important predictors of melanism than urbanization and park size. Interaction effects showed substantially lower importance weights compared to all primary environmental predictors

Table S1. Top 10 GLM dredge models by AICc. Canopy cover and winter temperature had large effect sizes and were significant across all models. 4 models (bold) are within 2 AICc units of the top model.

| Model Rank | Formula | AICc | ΔAICc | Residual Deviance |
| --- | --- | --- | --- | --- |
| **1** | Canopy Cover + Winter Temperature | 262.67 | 0 | 161.19 |
| **2** | Canopy Cover + Winter Temperature + Park Area | 264.07 | 1.4 | 160.58 |
| **3** | Canopy Cover x Winter Temperature | 264.1 | 1.43 | 160.62 |
| **4** | Canopy Cover + Urbanization Index + Winter Temperature | 264.2 | 1.53 | 160.72 |
| 5 | Canopy Cover + Urbanization Index + Winter Temperature + Park Area | 265.06 | 2.39 | 159.57 |
| 6 | Canopy Cover + Urbanization Index × Winter Temperature | 265.36 | 2.69 | 159.88 |
| 7 | Canopy Cover ×Winter Temperature + Urbanization Index | 265.57 | 2.9 | 160.08 |
| 8 | Canopy Cover × Winter Temperature + Park Area | 265.68 | 3.01 | 160.19 |
| 9 | Canopy Cover + Urbanization Index × Winter Temperature + Park Area | 265.63 | 2.96 | 158.15 |
| 10 | Canopy Cover + Winter Temperature + Park Area × Winter Temperature | 265.88 | 3.21 | 160.4 |
